# Downgrade of cardiac defibrillator devices to pacemakers in elderly heart failure patients: clinical considerations and the importance of shared decision-making

**DOI:** 10.1007/s12471-021-01555-w

**Published:** 2021-03-12

**Authors:** H. F. Groenveld, J. E. Coster, D. J. van Veldhuisen, M. Rienstra, Y. Blaauw, A. H. Maass

**Affiliations:** Department of Cardiology, Heart Centre, University Medical Centre Groningen, University of Groningen, Groningen, The Netherlands

**Keywords:** Heart failure, Implantable cardioverter defibrillator, Downgrade

## Abstract

Implantable cardioverter defibrillators are implanted on a large scale in patients with heart failure (HF) for the prevention of sudden cardiac death. There are different scenarios in which defibrillator therapy is no longer desired or indicated, and this is occurring increasingly in elderly patients. Usually device therapy is continued until the device has reached battery depletion. At that time, the decision needs to be made to either replace it or to downgrade to a pacing-only device. This decision is dependent on many factors, including the vitality of the patient and his/her preferences, but may also be influenced by changes in recommendations in guidelines. In the last few years, there has been an increased awareness that discussions around these decisions are important and useful. Advanced care planning and shared decision-making have become important and are increasingly recognised as such. In this short review we describe six elderly patients with HF, in whose cases we discussed these issues, and we aim to provide some scientific and ethical rationale for clinical decision-making in this context. Current guidelines advocate the discussion of end-of-life options at the time of device implantation, and physicians should realise that their choices influence patients’ options in this critical phase of their illness.

## Introduction

Heart failure (HF) remains a prevalent and major health care issue in the Western world, and this is strongly associated with the ageing population [1, 2]. The life expectancy of HF patients has also markedly increased because of successful evidence-based medical treatment, as well as the introduction of the implantable cardioverter defibrillator (ICD) and cardiac resynchronisation therapy (CRT). Since the introduction of cardiac implantable electronic devices almost 20 years ago, their use has increased enormously throughout Europe [3].

Although sudden death has always been one of the most common causes of death in HF, a recent large meta-analysis of HF trials showed that between 1995 and 2014 the rate of sudden death at 90 days declined by 44%, which was only partly due (in that study) to device therapy [4]. In addition, the Danish Study to Assess the Efficacy of ICDs in Patients with Non-ischemic Systolic Heart Failure on Mortality (DANISH) showed that ICD implantation did not reduce all-cause mortality in non-ischaemic cardiomyopathy patients [5]. Also, it has been shown that ICD implantation is probably less useful in reducing the incidence of sudden death in elderly patients (as compared to younger patients), since many older patients die of non-cardiovascular causes, and sudden cardiac death caused by ventricular arrhythmia is probably less common in these patients [6, 7]. In addition to these issues regarding the efficacy of the ICD, a small but significant proportion (9% at 5 years in a recent study) [8] deliver inappropriate shocks, which may be very stressful for patients. In the light of these findings, implantation of an ICD in elderly HF patients has become less automatic [9], and this is also true in patients who have an ICD (or CRT device with ICD function, CRT-D) in which the battery is approaching its end-of-life. In the last few years, a large study has been conducted in the Netherlands, which examines outcome measures and appropriate shocks of ICD therapy in HF patients [10], and the results of this trial are eagerly awaited.

In recent years it has become increasingly clear that these decisions can be very difficult and are preferably made by a multidisciplinary team. The current HF guidelines state that an experienced cardiologist should evaluate each patient before generator replacement, but specific recommendations regarding device replacement in the elderly and sometimes frail patients are lacking [1, 11].

In these discussions in elderly patients, clearly, not only medical considerations play a role, but all aspects of care for these patients must be integrated into what is called palliative care (which is certainly not the same as terminal or end-of-life care) [12]. Indeed, there is increased awareness that in elderly HF patients advanced care planning [13] but also shared decision-making with the patient [9, 14, 15] play an important role, also when considering device replacement.

**Fig. 1:**
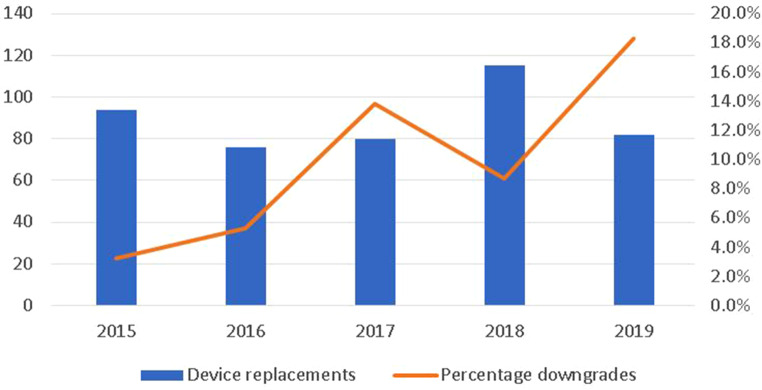
Number of implantable cardioverter defibrillator and cardiac resynchronisation therapy defibrillator replacements as well as the percentage of downgrades at the University Medical Centre in Groningen

In the present report we describe six elderly HF patients in whom device replacement because of battery end-of-life was indicated, and in whom we considered downgrading of this device. Through these case vignettes we aim to give a clinical perspective on device replacement in the ageing HF population. Fig. 1 shows the number of ICD replacements at our centre and the increasing percentage of downgrades. The relevance of the issue addressed is shown by the increasing number of downgrades.

## Case vignettes

*Patient 1* was an 84-year-old man who was seen in our outpatient clinic for HF associated with an ischaemic aetiology. He had undergone coronary artery bypass grafting (CABG) in 1999, when his left ventricular ejection fraction (LVEF) was 45%. In 2004 he received a dual-chamber pacemaker due to bradycardia-tachycardia syndrome. In 2010 he was hospitalised for decompensated HF. Coronary angiography showed open grafts and no culprit lesions. His LVEF had declined to 30%, and it was concluded that this could be related to continuous right ventricular (RV) pacing. His pacemaker was upgraded to a CRT‑D. In the following years he remained stable under optimal HF therapy. He received successful antitachycardia pacing (ATP) for ventricular tachycardia in 2013, and thereafter no further ATP episodes occurred. In 2017 his clinical condition declined due to progression of his HF (New York Heart Association (NYHA) class III–IV) and his quality of life decreased. His outpatient cardiologist had already discussed end-of-life issues, including possible discontinuation of his ICD, which was also related to the fact that his renal function was poor. When his CRT‑D reached the elective replacement interval the patient and outpatient cardiologist agreed to downgrade the CRT‑D to a CRT with only pacing mode (CRT-P). One and a half years later, the patient is doing reasonably well.

*Patient 2* was an 86-year-old man, who was referred to our tertiary centre for an ICD generator change, because of end-of-life of the battery. Outpatient follow-up was performed in a referral hospital. The patient underwent a CABG in 1998 and received an ICD in 2010 for primary prevention, as he was found to have a reduced LVEF of 30%. He has not received ICD therapy. He had developed progressive complaints of HF in 2014, and overall his vitality had declined over the years. His outpatient cardiologist discussed the pros and cons of a generator change, and also the option of a downgrade to a regular dual-chamber pacemaker, as he was known to have 97% atrial pacing before. By mutual consent it was decided to perform a downgrade to a pacemaker, and the procedure was uneventful.

*Patient 3* was an 80-year-old woman who was regularly seen in our outpatient clinic for non-ischaemic HF. She was known to have had dilated cardiomyopathy since 1995 and had very few complaints. Her LVEF varied between 35% and 45% between 1995 and 2011. In 2011, she developed more severe dyspnoea and fatigue, despite increased HF medication, and her LVEF decreased to 28%. In combination with the fact that she also had a left bundle branch block (LBBB), it was decided to implant a CRT‑D. The patient improved clinically, and her LVEF increased to 55%; her functional status improved from NYHA class III to NYHA I. She did not receive ATP for ICD shocks during the following years. In 2016 metastatic lung cancer was diagnosed, for which she received chemotherapy. Although she initially responded well, her condition deteriorated in 2018. Since her pacemaker also had to be replaced within the next few months, it was agreed to downgrade her device to a CRT‑P when the time arrived. However, the patient’s clinical status rapidly worsened, her pulmonary function declined, and the ICD shock function was discontinued. The patient died shortly thereafter.

*Patient 4* was a 77-year-woman who was referred to our tertiary centre in 2010 for screening because of a familial dilated cardiomyopathy. At the time she was asymptomatic, but an LBBB was found, as well as an LVEF of 45%. She received medical treatment, and her condition was stable until 2016 when she developed HF symptoms and her LVEF had declined to 30%. She received a CRT‑D and HF symptoms disappeared, while her LVEF improved to 60%. In 2019 she presented with 14 inappropriate ICD shocks due to noise caused by a fractured RV shock electrode. At that time it was decided to temporarily deactivate the ICD shock function and to programme the device to LV pacing only. Four weeks later, the patient presented with increasing shortness of breath, and LV pacing had decreased to 56% due to incessant noise from the RV lead. Three options were discussed with the patient: (1) placement of a new RV electrode (pacemaker or ICD electrode); (2) switching LV and RV electrodes on the current device and providing LV-only pacing via the RV channel; (3) downgrading to a pacemaker with LV-only pacing using a conventional DDD pacemaker programmed to fusion pacing with intrinsic right bundle activation. The treating cardiologist discussed the situation with her at length, and one point of consideration was, clearly, that over the past 3 years she had not needed the ICD, and no appropriate shocks had been delivered. Besides, she was very frightened of inappropriate ICD shocks and decided to choose option 3. Moreover, she was happy that there was no ICD function anymore.

*Patient 5* was a 90-year-old man who was known to have had a dilated cardiomyopathy for many years when a CRT‑D was implanted in 2013. He had been clinically stable for many years before, but in 2012 he had increasing HF complaints for which medication was started. His LVEF, which had been 50–55% in the past, had decreased to 32%. After CRT‑D implantation, he gradually improved clinically, and his LVEF increased to 40–45%. In the previous few years, he had been relatively active and was still very much enjoying his life. In the years after his CRT‑D implantation, he never had a shock or ATP episode. When his battery reached end-of-life, the option of downgrading the device was discussed, i.e. not replacing the CRT‑D but implanting a CRT‑P instead. It was explained to him that he had never had a shock and, also, that the guidelines regarding indications for ICD implantation had changed over the years, which meant a less strict (or even no) indication in his case. Nevertheless, he wanted to receive a CRT‑D; generator change was performed and was uneventful. Six months after CRT‑D replacement he was hospitalised for a sepsis, which resolved after antibiotics. During hospital admission, the ICD function of his CRT‑D was deactivated, at the patients’ request. One year later he is still living at home.

*Patient 6* was a 76-year-old man who was known to have had an inferoposterior infarct; his LVEF was 45%, and he had had permanent atrial fibrillation since 1998. In 2003 he was resuscitated owing to ventricular fibrillation, and no novel coronary lesions were detected. A single-chamber ICD was implanted, which was replaced in 2011 owing to battery depletion. In 2018 he was seen after 2 years without check-ups due to social problems, including severe grief over his wife’s death. The device showed one more year of battery life. The patient expressed the wish not be resuscitated anymore, since his quality of life had diminished dramatically. It was decided to explant his ICD when the device reached battery depletion. Device check-up demonstrated 35% RV pacing with programming set to VVI 70/min. The lower rate was decreased to 40/min and 1 month later to 30/min. Device check-up then demonstrated < 0.1% RV pacing, and the patient exhibited no symptoms related to bradycardia. Because it could not be ruled out that significant bradycardias were prevented by the low pacing percentage, RV output was reduced to subthreshold 0.1 V (pacing cannot be turned off in most ICDs). After no symptoms of bradycardia developed, explantation was subsequently performed.

Table 1 provides an overview of the various cases described above.

## Discussion

ICDs are used on a large scale in patients with HF and have been shown not only to be effective but also to reduce sudden death in many patients with HF with a reduced LVEF [1, 3]. These devices, i.e. ICDs alone or combined with CRT (CRT-D), have a class I recommendation in current HF guidelines, particularly in those patients with ischaemic heart disease [5, 7]. Nevertheless, a patient’s vitality but also his or her preferences will change over the years and, indeed, recommendations regarding indications for implantation may change as well, as was shown for patients with non-ischaemic cardiomyopathy [5]. As a result, replacement of an ICD/CRT‑D with the same device when its end-of-life is reached is not always automatic, particularly in the elderly. In this report we present six patients in whom this situation occurred. Regarding a defibrillator downgrade there are some specific considerations that need to be addressed.

### Disease trajectory of patients with HF

Patients with HF will experience a gradual deterioration in physical health over the years and, considering the progressive decline, the treatment goals will change over time. Given the general prognosis of HF it is unthinkable not to address end-of-life issues with elderly HF patients [12, 13, 15].

In elderly patients with an implantable device one should consider the pros and cons of ICD downgrade. This should be part of the regular clinical follow-up, but should definitely be discussed when the elective replacement interval is reached [11].

The annual sudden death rate in the HF population has substantially declined over the last 20 years due to better drug and device therapy [4]. In addition, the incidence rate of sudden death declines with increasing age [6, 7]. A study by Kinch Westerdahl et al. [16] analysed the incidence and relevance of ventricular arrhythmias and shocks of explanted ICDs of deceased patients. They examined 125 patients, of whom 93% had HF. The cause of death was non-arrhythmic in 75% of patients (progressive HF in 37% and non-cardiac in 38%). In addition, up to one-third of patients had ventricular tachyarrhythmia events leading to shocks close to death, and inappropriate shocks occurred in 13% of patients. The authors also reported that despite a do-not-resuscitate order, ICDs were often (> 50% of patients) not turned off, and patients received shocks during the last hours of life. Grubman et al. [17] also studied the stored electrograms in ICDs of deceased HF patients and concluded that 94% of the deaths were not the immediate result of a tachyarrhythmia.

### ICD recommendation for non-ischaemic cardiomyopathy

The current European Society of Cardiology (ESC) HF guidelines (2016) recommend an ICD implantation in all symptomatic non-ischaemic HF patients with an LVEF < 35% [1]. However, shortly after the publication of these ESC HF guidelines, the DANISH study was published [5]. This study in 1116 patients showed that prophylactic ICD implantation did not reduce all-cause mortality in patients with non-ischaemic HF during more than 5.5 years of follow-up. As a result, the strong recommendation in (all) patients with non-ischaemic cardiomyopathy and systolic HF has been questioned [18]. Interestingly, in a post hoc analysis of the DANISH study, it was shown that there was a linear decreasing relationship between ICD implantation and mortality with age [19]. While there was a survival benefit of the ICD in patients ≤ 70 years of age, no effect of the ICD was observed in patients > 70 years old [19].

### Responders to cardiac resynchronisation

About two-thirds of HF patients respond to CRT with an improvement in LV function and/or a reduction in ventricular volumes. This clinical improvement leads to a reduction in morbidity and mortality [1, 20]. Also, several studies have shown that this improvement leads to a reduced risk of ventricular arrhythmias [20, 21] and, in a large study, CRT (without an ICD) was associated with a significant and long-term reduction in sudden death [22]. Moreover, an increase in LVEF to > 40% after CRT would theoretically nullify the ICD indication, or at least this should be taken into consideration, since such an improvement in LV function markedly affects outcome [22–24].

A retrospective analysis in a real-life population from the Netherlands showed that patients with an LVEF > 35% were at low risk of ventricular arrhythmias [25]. Normalisation of LVEF with CRT reduces the risk of sudden cardiac death to a level comparable to that in the general population [23, 26]. Several favourable prognostic factors (i.e. female sex, LBBB, baseline LVEF > 30%) have been associated with a low risk of ventricular arrhythmias [21]. Nevertheless, despite improvement of LV function after CRT, identification of ‘low-risk’ patients remains difficult and so it is difficult to identify patients who may not ‘need’ an ICD (anymore) [24]. Despite the excellent prognosis in super-responders, ventricular arrythmias can still occur [27]. Thus the underlying substrate remains present and can trigger ventricular arrhythmias. In addition, underlying disease (coronary artery disease) can progress and reduce LV function.

### Shared decision-making, advanced care planning and palliative care

Patients with HF have a decreased life expectancy and quality of life [1], and while palliative care has been more generally accepted in other chronic illnesses, in particular cancer [28], the number of reports in HF has remained relatively small until recently [12]. In the last 10 years, however, this part of HF management has received increasing attention, and in the current ESC HF guidelines of 2016, palliative care and discussions on end-of-life decisions and deactivation of ICDs have also been included [1]. Initiating the discussion regarding a deactivation of an ICD, or downgrading a pacemaker device, may be very difficult, whereby patients’ insufficient knowledge about the disease and about the value of an ICD plays an important role [9, 12, 15, 29]. The issue of a (possible, future) downgrade should be addressed early and, ideally, this should be tackled when an ICD is first implanted. There may be several challenges to having such conversations, and indeed to delivering palliative care in general to HF patients, including prognostic uncertainty, timing of the discussion, i.e. too late initiation of this trajectory, ambiguity concerning the coordination of care, difficulties in communication on both sides, and discrepant expectations of patients/family, preferences, needs and values [30]. There is convincing evidence, however, that palliative care and advanced care planning are crucial, and reduce symptoms and hospitalisations, as well as improving quality of life [13, 30, 31].

**Fig. 2:**
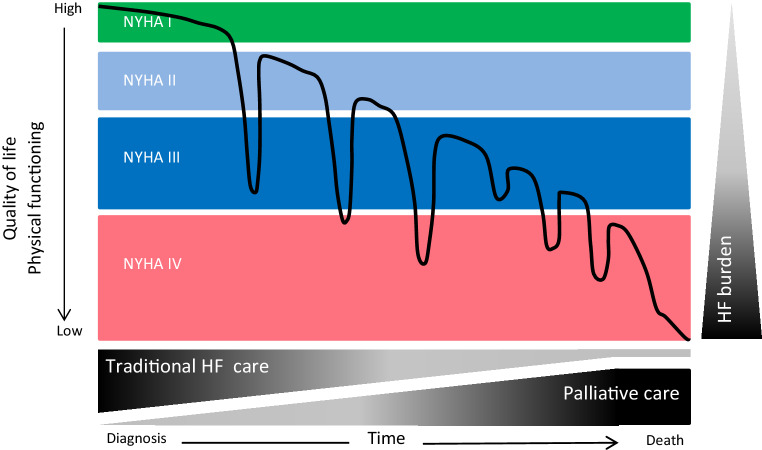
Disease trajectory and transition in heart failure care. *NYHA* New York Heart Association class, *HF* heart failure

As the patient with HF deteriorates over time (Fig. 2), and this particularly holds when the device is up for replacement (because of battery end-of-life), such discussions should always take place. This may not be easy though, and a recent survey from Sweden from 2018 [15] showed that up to 40% of patients who had an ICD (with or without HF) at no point wanted to discuss their disease trajectory. Moreover, only patients who had experienced an ICD shock, or who had high levels of anxiety, were prepared to have such discussions with their health care providers.

**Fig. 3:**
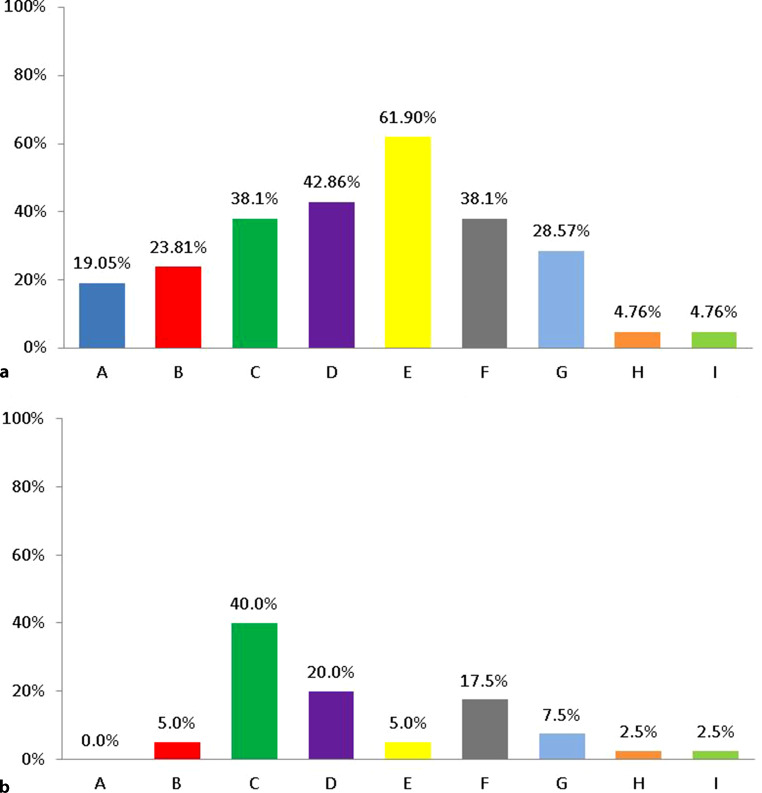
**a** When replacing a cardiac resynchronisation therapy defibrillator (CRT-D), in which of the following cases would you downgrade to a cardiac resynchronisation therapy pacemaker (CRT-P)? Adapted from [11], with permission. **b** Cases at the University Medical Centre in Groningen downgraded to a non-defibrillator device. Possible reasons to downgrade a CRT‑D to a CRT‑P device: *A* never; *B* left ventricular ejection fraction (LVEF) > 40%, ischaemic cardiomyopathy without appropriate device therapies; *C* LVEF > 40%, non-ischaemic cardiomyopathy without appropriate device therapies; *D* irreversible severe heart failure (New York Heart Association class IV); *E* severe medical condition (e.g. neoplasia) with < 1 year life expectancy; *F* advanced age (e.g. > 80 years); *G* frailty; *H* prior inappropriate ICD therapies and no appropriate therapies since implantation, regardless of cardiomyopathy; *I* no appropriate therapies, high risk of inappropriate therapy (e.g. development of atrial fibrillation)

Downgrading a device, specifically not replacing the ICD at the end of its battery life, however, does not often occur, and was recently shown in a large study to be done in less than 10% of cases [11]. Replacing the CRT‑D device with a CRT (P) (i.e. not having the ICD function anymore) may be considered in several clinical situations, however. In this same large European survey [11], a patient’s life expectancy < 1 year was the most common reason for a downgrade (62%), followed by irreversible severe HF (42%) and advanced age, i.e. > 80 years old (38%), while frailty was also mentioned in 28% (Fig. 3a). LVEF > 40% and non-ischaemic cardiomyopathy without appropriate device therapies was the most common reason for downgrading the ICD in our cases (Fig. 3b).

Advanced age is maybe the most difficult issue in these considerations. The current ESC HF guidelines do not mention age [1], but clearly frailty and decreased life expectancy will play a role in some patients. Also, there is evidence, as discussed above, that the value of an ICD in HF is markedly less in elderly patients, which may also have to do with the fact that older HF patients have many comorbidities, and much less often die from cardiovascular causes, let alone sudden cardiac death caused by ventricular arrhythmia. Calendar or chronological age is certainly not the only parameter here, and ‘functional age’ is obviously more important [28], but this is not always easy to quantify. In our small series, all patients were of advanced age but, remarkably, the oldest patient was the only one who did not agree to downgrading of his device.

### Technical considerations

Downgrading a DF-1/IS‑1 connector is, of course, not an issue. However, no adapters are available for a DF‑4 connector. Thus, in the case of a DF‑4 connector a true downgrade can only be performed by implanting a new RV lead. An artificial downgrade is, of course, possible by turning off the tachycardia therapies in the new device. This is, however an expensive solution. In addition, ICD devices have fewer programming options than pacing-only devices. For example, LV-only pacing is possible but not without RV sensing. Considering that it is not possible to downgrade DF‑4 connectors we prefer the DF-1/IS‑1 connector at the time of device implantation. This is in contrast with current practice in which > 90% of the implanted RV leads are DF‑4 [32].

## Conclusions

Downgrading of device therapy in elderly patients with HF at the time of pacemaker replacement, i.e. discontinuation of the ICD part, must be discussed and considered at the time of pacemaker replacement or battery end-of-life. ICDs are less useful in elderly patients, and patients with non-ischaemic cardiomyopathy/HF may also derive less benefit. CRT alone (without ICD) improves LV function and may be considered in some elderly patients. Shared decision-making between doctor and patient and advanced care planning are crucial and will help increase understanding and facilitate ‘tailor-made therapy’ according to the wishes of the HF patients, thereby helping to resolve any issues that may be involved.

**Table 1:** Overview of cases

Patient	Sex	Age (years)	HF aetiology	ICD type	ICD prevention	Downgrade	Considerations	Replacement device
*1*	Male	84	Ischaemic	CRT	Primary	Yes	Clinical deterioration of HF	*CRT‑P*
*2*	Male	86	Ischaemic	DDD	Primary	Yes	Reduced vitality, no history of ICD therapy	*DDD-PM*
*3*	Female	80	Nonischaemic	CRT	Primary	Yes	Malignancy with distant metastasis	*CRT‑P*
*4*	Female	77	Nonischaemic	CRT	Primary	Yes	Multiple inappropriate ICD shocks due to RV lead failure	*DDD-PM* *(LV-only pacing)*
*5*	Male	90	Nonischaemic	CRT	Primary	No	Patient’s preference for CRT‑D replacement	*CRT‑D*
*6*	Male	76	Ischaemic	VVI	Secondary	Yes	Low quality of life, do-not-resuscitate order among patient’s preferences	*Explantation*

*HF* heart failure, *ICD* implantable cardioverter defibrillator, *RV* right ventricular, *CRT‑D* cardiac resynchronisation therapy defibrillator, *CRT‑P* cardiac resynchronisation therapy pacemaker, *DDD-PM* DDD pacemaker, *LV* left ventricular

## References

[1] Ponikowski P, Voors AA, Anker SD (2016). 2016 ESC guidelines for the diagnosis and treatment of acute and chronic heart failure. Eur Heart J.

[2] Ferreira JP, Metra M, Mordi I (2019). Heart failure in the outpatient versus inpatient setting: findings from the BIOSTAT-CHF study. Eur J Heart Fail.

[3] van Veldhuisen DJ, Maass AH, Priori SG (2009). Implementation of device therapy (cardiac resynchronisation therapy and implantable cardioverter defibrillator) for patients with heart failure in Europe: changes from 2004 to 2008. Eur J Heart Fail.

[4] Shen L, Jhund PS, Petrie MC (2017). Declining risk of sudden death in heart failure. N Engl J Med.

[5] Kober L, Thune JJ, Nielsen JC (2016). Defibrillator implantation in patients with nonischemic systolic heart failure. N Engl J Med.

[6] Krahn AD, Connolly SJ, Roberts RS, Gent M (2004). Diminishing proportional risk of sudden death with advancing age: implications for prevention of sudden death. Am Heart J.

[7] Coiro S, Girerd N, Sharma A (2019). Association of diabetes and kidney function according to age and systolic function with the incidence of sudden cardiac death and non-sudden cardiac death in myocardial infarction survivors with heart failure. Eur J Heart Fail.

[8] Mattsson G, Magnusson P (2020). Long-term follow-up of implantable cardioverter defibrillator patients with regard to appropriate therapy, complications, and mortality. Pacing Clin Electrophysiol.

[9] Hess PL, Matlock DD, Al-Khatib SM (2020). Decision-making regarding primary prevention implantable cardioverter-defibrillators among older adults. Clin Cardiol.

[10] van Barreveld M, Dijkgraaf MGW, Hulleman M (2017). Dutch outcome in implantable cardioverter-defibrillator therapy (DO-IT): registry design and baseline characteristics of a prospective observational cohort study to predict appropriate indication for implantable cardioverter-defibrillator. Neth Heart J.

[11] Tilz R, Boveda S, Deharo J, Dobreanu D, Haugaa KH, Dagres N (2016). Replacement of implantable cardioverter defibrillators and cardiac resynchronization therapy devices: results of the European heart rhythm association survey. Europace.

[12] Kida K, Doi S, Suzuki N (2020). Palliative care in patients with advanced heart failure. Heart Fail Clin.

[13] Schichtel M, Wee B, Perera R, Onakpoya I (2020). The effect of advance care planning on heart failure: a systematic review and meta-analysis. J Gen Intern Med.

[14] Fried TR (2016). Shared decision making—finding the sweet spot. N Engl J Med.

[15] Thompson JH, Thylen I, Moser DK (2019). Shared decision-making about end-of-life care scenarios compared among implantable cardioverter defibrillator patients: a national cohort study. Circ Heart Fail.

[16] Kinch Westerdahl A, Sjöblom J, Mattiasson A, Rosenqvist M, Frykman V (2014). Implantable cardioverter-defibrillator therapy before death: high risk for painful shocks at end of life. Circulation.

[17] Grubman EM, Pavri BB, Shipman T, Britton N, Kocovic DZ (1998). Cardiac death and stored electrograms in patients with third-generation implantable cardioverter-defibrillators. J Am Coll Cardiol.

[18] Akhtar M, Elliott PM (2019). Risk stratification for sudden cardiac death in non-ischaemic dilated cardiomyopathy. Curr Cardiol Rep.

[19] Elming MB, Nielsen JC, Haarbo J (2017). Age and outcomes of primary prevention implantable cardioverter-defibrillators in patients with nonischemic systolic heart failure. Circulation.

[20] Sebag FA, Lellouche N, Chen Z (2014). Positive response to cardiac resynchronization therapy reduces arrhythmic events after elective generator change in patients with primary prevention CRT-D. J Cardiovasc Electrophysiol.

[21] Ruwald MH, Solomon SD, Foster E (2014). Left ventricular ejection fraction normalization in cardiac resynchronization therapy and risk of ventricular arrhythmias and clinical outcomes: results from the multicenter automatic defibrillator implantation trial with cardiac resynchronization therapy (MADIT-CRT) trial. Circulation.

[22] Cleland JG, Daubert JC, Erdmann E (2006). Longer-term effects of cardiac resynchronization therapy on mortality in heart failure [the CArdiac REsynchronization-Heart Failure (CARE-HF) trial extension phase. Eur Heart J.

[23] Manne M, Rickard J, Varma N, Chung MK, Tchou P (2013). Normalization of left ventricular ejection fraction after cardiac resynchronization therapy also normalises survival. Pacing Clin Electrophysiol.

[24] Narducci ML, Biffi M, Ammendola E (2018). Appropriate implantable cardioverter-defibrillator interventions in cardiac resynchronization therapy-defibrillator (CRT-D) patients undergoing device replacement: time to downgrade from CRT-D to CRT-pacemaker? Insights from real-world clinical practice in the DECODE CRT-D analysis. Europace.

[25] ter Horst IA, van’t Sant J, Wijers SC, Vos MA, Cramer MJ, Meine M (2016). The risk of ventricular arrhythmias in a Dutch CRT population: CRT-defibrillator versus CRT-pacemaker. Neth Heart J.

[26] Manfredi JA, Al-Khatib SM, Shaw LK (2013). Association between left ventricular ejection fraction post-cardiac resynchronization treatment and subsequent implantable cardioverter defibrillator therapy for sustained ventricular tachyarrhythmias. Circ Arrhythm Electrophysiol.

[27] Zecchin M, Proclemer A, Magnani S (2014). Long-term outcome of ‘super-responder’ patients to cardiac resynchronization therapy. Europace.

[28] Soto-Perez-de-Celis E, Li D, Yuan Y, Lau YM, Hurria A (2018). Functional versus chronological age: geriatric assessments to guide decision making in older patients with cancer. Lancet Oncol.

[29] Stromberg A, Fluur C, Miller J, Chung ML, Moser DK, Thylen I (2014). ICD recipients’ understanding of ethical issues, ICD function, and practical consequences of withdrawing the ICD in the end-of-life. Pacing Clin Electrophysiol.

[30] Chow J, Senderovich H (2018). It’s time to talk: challenges in providing integrated palliative care in advanced congestive heart failure. a narrative review. Curr Cardiol Rev.

[31] Kernick LA, Hogg KJ, Millerick Y, Murtagh FEM, Djahit A, Johnson M (2018). Does advance care planning in addition to usual care reduce hospitalisation for patients with advanced heart failure: a systematic review and narrative synthesis. Palliat Med.

[32] Li JZ, Bhonsale A, Estes NAM (2019). Trends and implications of DF-4 implantable cardioverter-defibrillator lead adoption in the United States of America. Circ Arrhythm Electrophysiol.

